# Functional Materials Based on Active Carbon and Titanium Dioxide in Fog Seal

**DOI:** 10.3390/ma13225267

**Published:** 2020-11-21

**Authors:** Chuan He, Qingyi Xiao, Fangyuan Gong, Yun Yang, Xipeng Ren

**Affiliations:** 1 School of Civil and Transportation Engineering, Hebei University of Technology, Tianjin 300401, China; 201821602019@stu.hebut.edu.cn (C.H.); fgong1@mtu.edu (F.G.); 201931604006@stu.hebut.edu.cn (X.R.); 2 Administration Office, Tianjin Yongyang Highway Engineering Group Co., Ltd., Tianjin 301700, China; wqjtjyyzhb@tj.gov.cn

**Keywords:** titanium dioxide, photocatalysis, asphalt concrete pavement, active carbon, fog seal

## Abstract

Due to its ability to degrade nitrogen oxides under ultraviolet, titanium dioxide has been applied in asphalt concrete to degrade automobile exhaust in recent years. To highlight the protection of road traffic environmental quality and mitigate automobile exhaust on human health, this study proposes combining titanium dioxide and active carbon into Sand-fog seal to form a pavement coating material with a photocatalytic function. It uses active carbon to reinforce the material’s function, and the coupling agent for modification makes it well dispersed in the Sand-fog seal. The indoor experiments were carried out at 30 °C and relative humidity of 30%. It tested the composite material’s degradation efficiency on nitrogen dioxide in relation to component proportions, coupling agents, and dosages. The study concluded that the optimal photocatalytic efficiency could be achieved when the ratio of active carbon to titanium dioxide is 0.6. After being modified by the titanate coupling agent and through Scanning Electron Microscope tests, it can be seen that materials can be well dispersed into the Sand-fog seal. When the composite material accounts for 10% of the fog seal, it can achieve the optimal photocatalytic efficiency of about 23.9%. The British pendulum tests show it has good skid resistance performance. Half a kilometer of concrete roadway was sprayed with the material coating in Tianjin, China. The photocatalytic experimental road degrades nitrogen oxides better than the original road. The method is feasible for practical implementation.

## 1. Introduction

In urban road traffic, nitrogen oxides (NO_x_) emissions from vehicle exhaust coexist with people in a specific area [1,2]. They are inhaled by the human body and affect the physical and mental health of people. Therefore, mitigating exhaust emissions has become the focus of protecting road traffic environmental quality [3,4]. Studies have found that titanium dioxide (TiO_2_) as a photocatalyst can significantly increase pollutants’ degradation efficiency. When nano-titanium dioxide is added to asphaltic materials, titanium dioxide will become a photocatalyst under the light. The hydrocarbon (HC) and NO_x_ of automobile exhaust will be catalytically decomposed into the corresponding carbonate and nitrate, which are adsorbed in the pavement’s texture and washed away [5,6]. In addition, titanium dioxide has good chemical stability compared with semiconductor photocatalyst. The principle of photocatalytic decomposition of automobile exhaust is illustrated in the following Formulas (1)–(3):

(1) $$CO+O_{2}\overset{UV,\;\mathrm{Nano}-{\mathrm{TiO}}_{2}}{\to }CO_{2}$$

(2) $$HC+O_{2}\overset{UV,\;\mathrm{Nano}-{\mathrm{TiO}}_{2}}{\to }H_{2}O+CO_{3}^{2-}$$

(3) $$NO_{X}+O_{2}\overset{UV,\;\mathrm{Nano}-{\mathrm{TiO}}_{2}}{\to }NO_{3}^{-}$$

The pavement surface is an excellent carrier with bearing nano-titania, as it can get enough ultraviolet (UV) light and contacts with exhausts directly. In addition, asphalt concrete undertakes most of the road pavement engineering. The combination of titanium dioxide and asphalt concrete pavement is also one of the focuses. Therefore, experts have introduced titanium dioxide as a photocatalytic material to cement concrete pavement and asphalt concrete pavement [7,8,9]. There are currently three main methods for adding titanium dioxide to asphalt concrete. Hassan et al. [1] coated titanium dioxide solution on the surface of asphalt concrete pavement. The results show evidence of a photocatalytic reaction occurring in the field, and the degradation rate of nitrogen oxide could reach 31–55%. Leng et al. [10,11] mixed titanium dioxide and aggregate to make asphalt concrete pavement. However, the degradation effect obtained by this method is limited. Another method is to put titanium dioxide into pavement maintenance materials and apply it to the surface of asphalt concrete [12]. However, the above method has the problem of low photocatalytic degradation efficiency, or it cannot readily achieve engineering practice. In addition, pure titanium dioxide cannot play its ideal functional performance compared with composites.

Scholars doped titanium dioxide with other materials such as carbon (C) to enhance the photocatalytic reaction. For instance, Rowan Leary and Aidan Westwood summarize the studies for composite materials such as C-TiO_2_ [13]. Active carbon is a porous carbonaceous matter with a highly developed pore structure that provides a large surface area. The huge surface area can have full contact with gas (impurities), thereby giving active carbon a unique absorptive property. That makes it very easy to absorb and collect impurities [14,15,16]. It can guide impurities to the surface of titanium dioxide for photocatalysis. In addition, active carbon can absorb the pungent odor in the exhaust, such as hydrocarbon (HC) and sulfur dioxide (SO_2_) to reduce the probability of inhalation. Therefore, this kind of composite material is highly concerned. However, these two materials do not readily dissolve in any solvent, so they need to be modified.

The modification of titanium dioxide is to amend its chemical and physical properties to improve the adsorption and degradation ability [17]. The methods to improve the dispersion of nanopowder in an organic phase medium are organic surface modification, mainly including polymer coating method [18], surfactant method and coupling agent method [19,20]. In the engineering process, chemical methods are applied to treat titanium dioxide. Among them, it is common to use the coupling agent to modify the powder. The coupling agent has two functional groups, including the hydrophilic group and the lipophilic group. The hydrophilic group in the molecule reacts with the hydroxyl group on the inorganic powder’s surface, which changes the property of its surface from hydrophily to lipophilicity. It improves the compatibility between inorganic powder and organic phase liquid (asphalt) [19]. In addition, the Sand-fog seal is mainly composed of emulsified asphalt and sand. This material often fills the tiny cracks and voids to prevent surface water from seeping [20,21]. Such material is often coated on the road surface directly contacting with sunlight. Thus, it can be used as a suitable carrier of photocatalysis [12]. Compared with other seals, the Sand-fog seal only contains small particles such as sand or clay, and its paving thickness is thinner than the other [22,23,24]. It required less titanium dioxide to achieve the photocatalytic reaction. Thus, the method of adding titanium dioxide to fog seal is an economical approach to engineering practice.

In this research, titanium dioxide is combined with carbon to make a photocatalytic composite material. Furthermore, it is devoted to preparing the composite fog seal material of titanium dioxide and carbon to find its optimal photocatalytic efficiency. Indoor experiments were carried out to achieve this objective. By using polytetrafluoroethylene (PTFE) to create the photocatalytic reaction mold, the photocatalytic efficiency was calculated by the difference of nitrogen dioxide concentration through the reaction. Since titanium dioxide and active carbon are not readily soluble in any solvent, they need to modified in advance. We compared the modifier, the ratio between carbon and titanium dioxide, and the fog seal’s dosage to find an optimal photocatalytic fog seal material. The experiments also compared the slip resistance between such materials and fog seal. In the outdoor experiment, we put such materials on an experimental road to detect the photocatalytic results. For the first time, this research proposes combining active carbon and titanium dioxide loaded on the road.

## 2. Indoor Experiments

### 2.1. Material Characterization

The experiments use anatase nano titanium dioxide (30 nm), density 2.45, and its technical indicators are shown in Table 1. Such materials are produced by Shanghai St-Nano Science and Technology Co., Ltd., Shanghai, China. Secondly, the experiment uses active carbon doped with titanium dioxide, and the specific indicators are shown in Table 2. The wood powder active carbon is manufactured by Henan Haixing Water Supply Materials Co., Ltd., Gongyi, China. The photocatalytic composite material is added to the Sand-fog seal (hereinafter referred to as fog seal) and then applied to the road. The Sand-fog seal used in this study was produced by Beijing Sealmaster, Ltd., Beijing, China. Table 3 shows its technical indicators. The experiments select four kinds of different coupling agents. It depends on the composition of functional groups. The models are KH550, KH570, KH792, and TCA (titanate coupling agent). The information of the four coupling agents is shown in Table 4. All coupling agents are produced by the Guangdong lvwei new materials technology, Ltd., Dongguan, China.

### 2.2. Testing System

**Figure 1:**
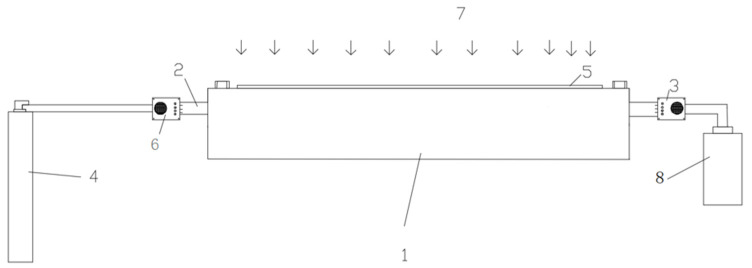
Photocatalytic system. (1) Reaction mold; (2) Running-in interface; (3) Probe; (4) Nitrogen dioxide bottle; (5) Cover plate; (6) Flow rate control; (7) Xenon lamp; (8) Waste bottle.

**Figure 2:**
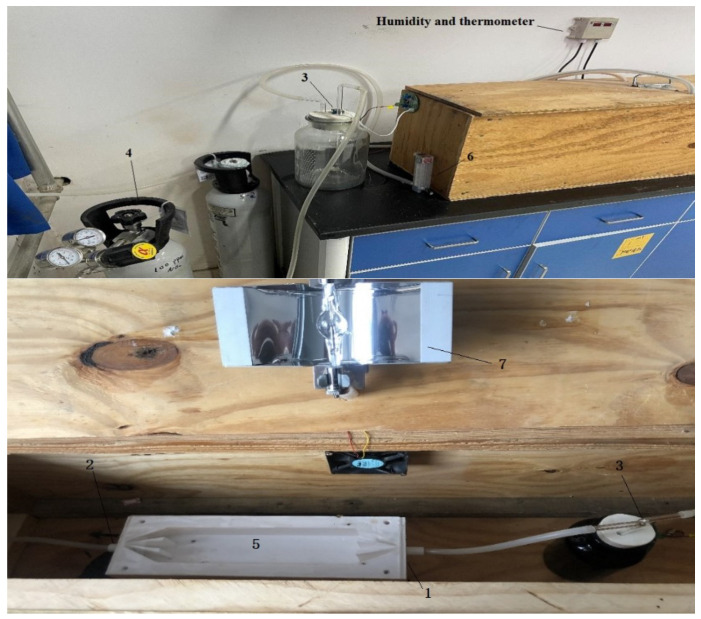
Test system; external (above), internal (below). (1) Reaction mold; (2) Running-in interface; (3) Probe; (4) Nitrogen dioxide bottle; (5) Cover plate; (6) Flow rate control; (7) Xenon lamp.

The photocatalytic reaction system is shown in Figure 1. The space formed between the multiple partitions serves as a gas input and output channel and enables the gas to reach a laminar flow state in the reactor closer to the actual emissions. The reactor and the partition are made of PTFE. Such material is resistant to acids, alkalis various organic solvents, and is almost insoluble in all solvents. At the same time, PTFE has the characteristics of high-temperature resistance. The size of the reactor is 300 mm × 100 mm × 50 mm. The diameter of the inlet and outlet of the reactor are both 10 mm. The cover plate is made of quartz glass with light transmission and high-temperature resistance. At both ends of the reactor, multiple partitions are applied. The photocatalytic materials are coated on a glass sheet and placed in the core of the mold. The concentration probe evaluates the NO_2_ degradation at the exit. The dimensions of the glass sheet are 200 mm × 50 mm × 2 mm. Such mold is connected with a quartz glass cover with glass glue to ensure no air leakage. The link on the left side of the photocatalytic mold is a nitrogen dioxide source with a concentration of 100 ppm (as shown in Figure 2). The mold’s right side is connected to the pipe and passed through the concentration probe and excluded from the outdoor.

### 2.3. Testing Principle

**Figure 3:**
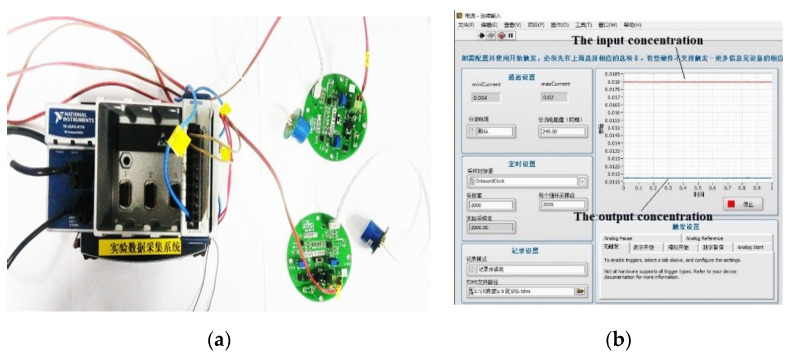
Testing probe (**a**) and data collection system (**b**).

The mold’s end is equipped with a specially customized nitrogen dioxide concentration probe (as shown in Figure 3a). The probe converts the concentration of nitrogen dioxide molecules into the current. The maximum current is 0.02 (100% concentration) and the minimum is 0.004 (0 concentration). It is connected to the computer, and the degradation of nitrogen dioxide is judged by the current difference before and after the reaction (as shown in Figure 3b). The detection system is shown in Figure 3. Before the experiment, the glass plate carrying the photocatalytic material is put into the reaction mold. The quartz glass cover plate’s edge was coated with glass glue to adhere to the mold. The mold is transferred into a wooden box with a xenon lamp on the wooden box cover. Before the experiment, we turned off the xenon lamp, and the wooden box cover was shut. Then, we opened the nitrogen dioxide gas. When the probe reached 100% concentration (current value is 0.02), the current stabilized, and we turned on the xenon lamp. The reaction began for a while, and the concentration began to drop (as the current drops). After falling to a certain degree of stability, we turned off the light, and the density rose. Through the maximum and minimum concentration (current) difference, experiments were repeated to calculate the amount of degradation. The degradation efficiency of nitrogen dioxide is shown in the following Formula (4);

(4) C%=(0.02−A1)/0.016

C%: Degradation efficiency A1: Current after stabilization.

### 2.4. Photodegradation Process

#### 2.4.1. Experimental Background

Environmental factors affect the photocatalytic efficiency, such as the humidity, flow rate, temperature and light density [25,26]. The maximum photocatalytic efficiency can be obtained in previous studies in which the reaction humidity is between 20 and 30% [27]. In addition, when the temperature is higher, the reaction speed is faster. Therefore, this study was carried out at room temperature of 30 degrees Celsius and humidity of 30%. The nitrogen dioxide flow is set to 1 L/min. In this experiment, titanium dioxide was added to the fog seal, which contains impurities and uneven particle sizes. The coating method, which uses a brush to apply a certain amount of composite material to the glass, is used for this experiment.

#### 2.4.2. Selection of Photocatalytic Composite

**Figure 4:**
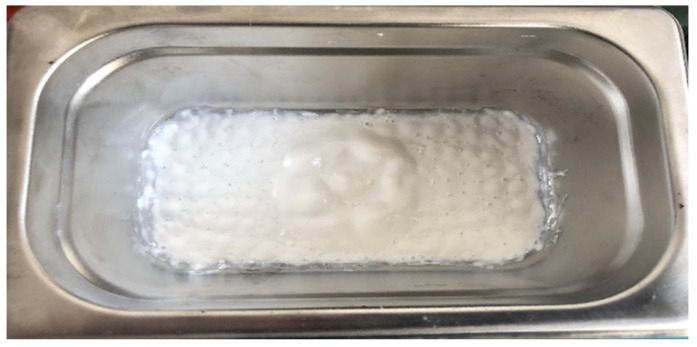
Ultrasonic vibration of titanium dioxide.

**Figure 5:**
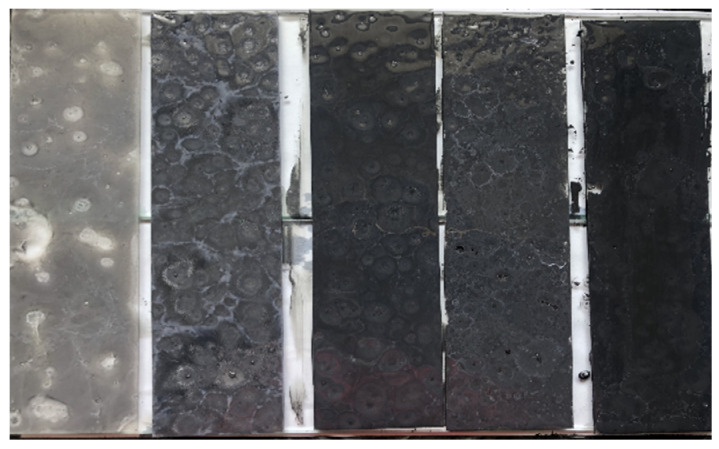
Composite materials in different proportions (from left to right: 0.1, 0.3, 0.6, 0.5, 0.7).

The experiments were divided into seven control variable groups to find the optimal combination effect. The weight ratio of active carbon and titanium dioxide was 0, 0.1, 0.3, 0.5, 0.6, 0.7, and 1, respectively (as shown in Table 5). Each sample was coated with the same amount of titanium dioxide, and the comparison was made by changing the amount of active carbon. The mixed solution is made from water as a carrier, coated on a glass sheet, and put into the mold for testing. We put 1 g of titanium dioxide and a certain amount of active carbon into the ultrasonic vibration instrument (as shown in Figure 4). Then, we added water as a carrier for ultrasonic vibration. Then, we applied it to a glass sheet covering an area of 0.01 m^2^ (as shown in Figure 5). Then, we put the glass sheet into the mold’s center, and the upper edge of the mold was coated with glass glue to adhere it to the glass plate. The cover was closed, the data were recorded when the current stabilized, and we turned on the xenon lamp to begin to react.

#### 2.4.3. Modifier for Composite

The photocatalytic materials were added to the fog seal through four coupling agents: KH550, KH570, KH792, and titanate coupling agent (TCA). The ratio of the composite in Section 2.4.2 was used. We mixed active carbon and titanium dioxide and then added absolute ethanol and water into ultrasonic to vibration. Then, 5 g coupling agents were mixed with fog seal material into such a solution, and they were ultrasonically vibrated for 10 min while being stirred. After being coated on glass plates, we left them to dry in the shade. It should be mentioned that the coupling agents were not easy to dry compared to water as the carrier in a short time. Therefore, it required enough time for the moisture to evaporate after being added to the fog seal. It takes at least eight hours to stop the degradation efficiency error caused by the dissolution of nitrogen dioxide into water or the coupling agent.

#### 2.4.4. Composite Dosage for Fog Seal

During pavement maintenance, fog seal is usually sprayed on the road surface to protect the road surface. In engineering, the coating amount of fog seal material on the road is usually 1–2 kg/m^2^. After ratio conversion (15 g/0.01 m^2^), we mixed a small amount of fog seal material with different photocatalyst dosages for the experiments. We used the optimal composite material ratio in Section 2.4.2. The optimal coupling agents were modified and added to the 15 g fog seal. After coating on a glass sheet, the specimen was left to dry. Then, we put it in the mold to start the reaction.

## 3. Pavement Performance

### 3.1. Skid Resistance Performance

**Figure 6:**
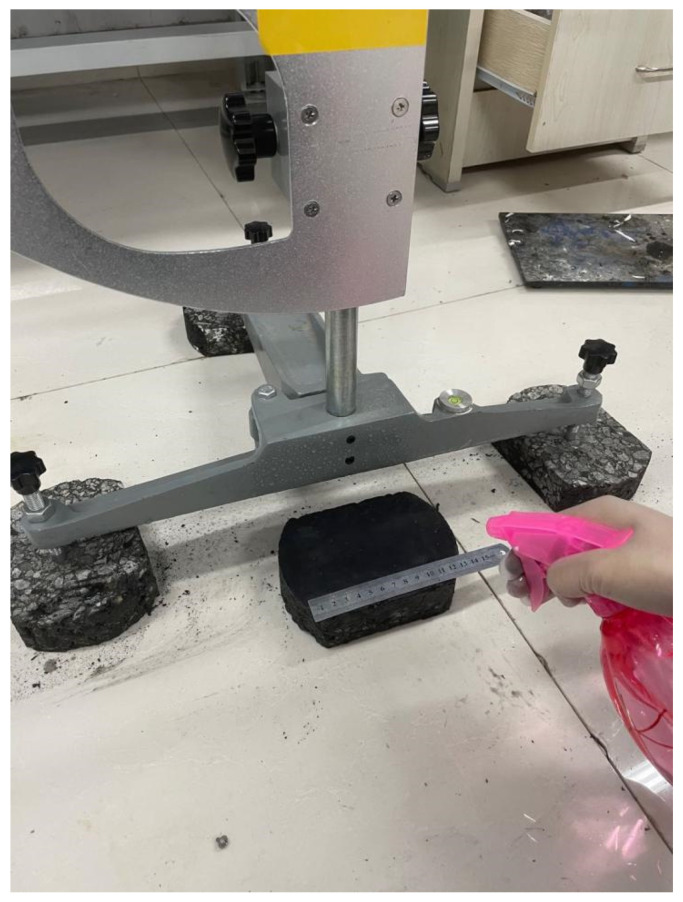
British pendulum tester.

**Figure 7:**
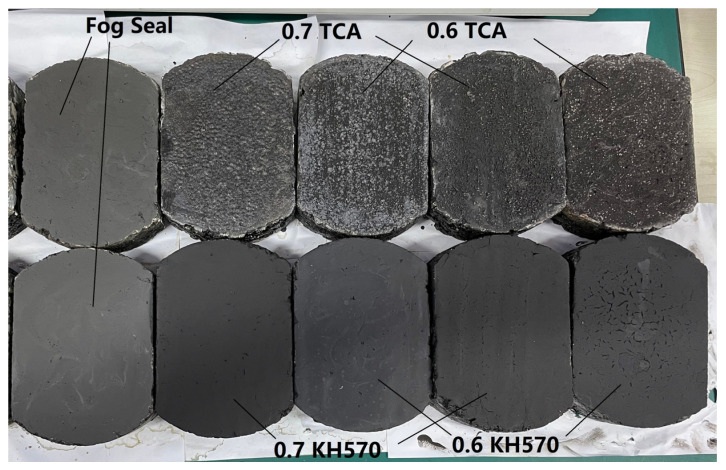
British pendulum number (BPN) samples prepared under different material ratios and coupling agent.

The British pendulum tests were also conducted in the laboratory (as shown in Figure 6). The specimens are made of different material and coupling agent ratios (as shown in Figure 7). The composite dosage is 10% while the fog seal is 15 g. The four combinations are based on the performance of photocatalytic efficiency and road performance in Section 2.4.

### 3.2. Nitrogen Oxide Detection

**Figure 8:**
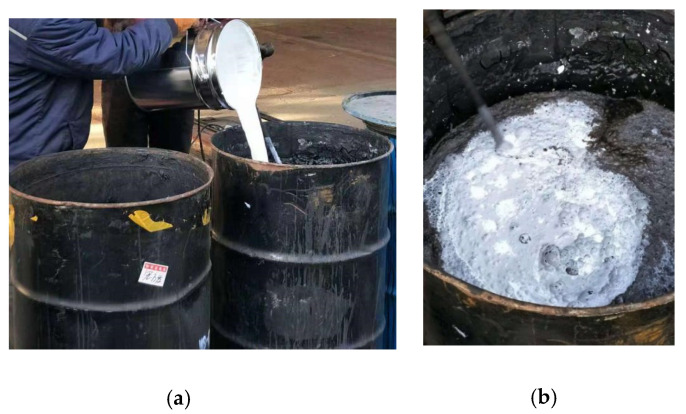
Preparation of functional Sand-fog seal: (**a**) adding; (**b**) mixing.

**Figure 9:**
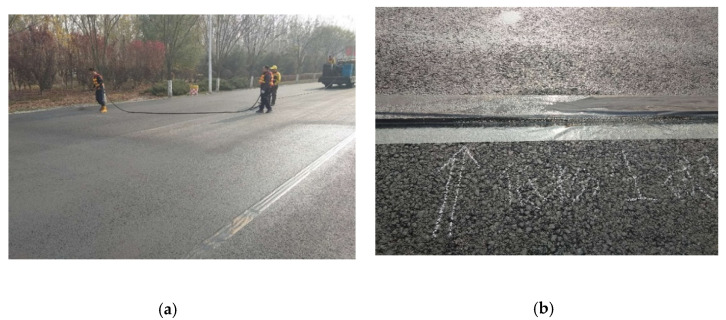
Spreading of functional Sand-fog seal: (**a**) Spraying; (**b**) Spray complete.

**Figure 10:**
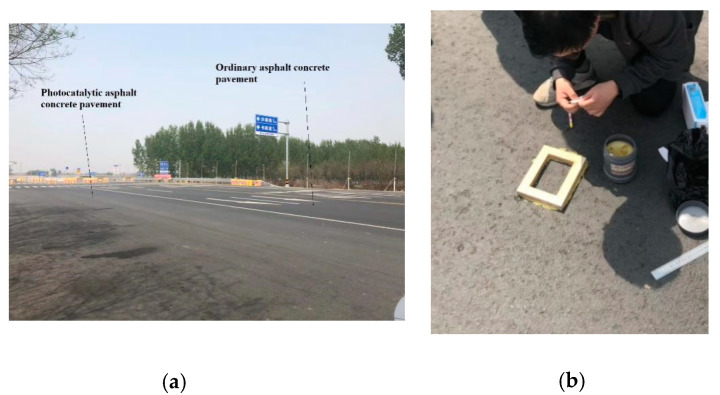
Outdoor nitrogen oxide collection experiment: (**a**) photocatalytic pavement and original pavement; (**b**) collecting water samples.

Through the photocatalytic efficiency and skid resistance performance of the composite material, we selected a material ratio that is most suitable for road paving. Then, such an amount of spreading for engineering practice was used to prepare for outdoor experiments (as shown in Figure 8 and Figure 9). The experiment road is located in Wuqing District, Tianjin, China. The composite was sprayed on the road, which had been completed but not yet opened to traffic (as shown in Figure 10). In the outdoor road test, the amount of nitrogen oxide was collected to determine the degradation effect. The method was mentioned in previous studies by Hassan et al. [1,25,26]. By placing a small amount of water on the road surface, the nitrogen oxides in the water were detected (as shown in Figure 10b). We compared the nitrogen oxide content on the photocatalytic road and ordinary roads (as shown in Figure 10a).

## 4. Results

### 4.1. Removal of NO_2_

**Figure 11:**
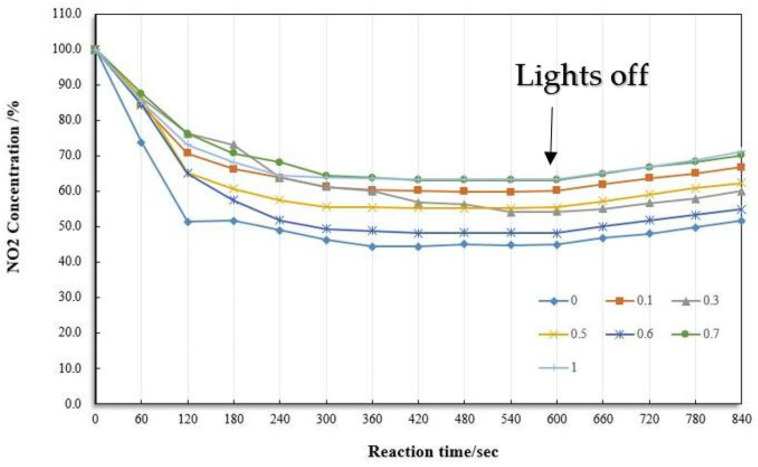
NO_2_ degradation efficiency under different Active Carbon/TiO_2_ ratios.

We compared seven composite materials with different ratios. After the experiment, the degradation efficiency of nitrogen dioxide is shown in Figure 11. It can be seen that the maximum degradation rate of nitrogen dioxide is about 54%, 39.9%, 43.8%, 44.9%, 51.9%, 36.9%, and 36.6%, respectively. These differences in the decomposition efficiency of nitrogen dioxide only originate from the carbon content. It can be seen that pure titanium dioxide had the highest speed degradation rate and a maximum photocatalytic efficiency. In previous studies, nitric oxide’s degradation experiment showed that the optimal ratio of active carbon to titanium dioxide was 0.7. The research also mentioned that the addition of active carbon spoiled the photocatalytic reaction [28]. Through the degradation of nitrogen dioxide in this experiment, it came to a similar conclusion. It is important to mention that when the ratio of active carbon to titanium dioxide is 0.6, such a degradation efficiency is slightly lower than that of pure titanium dioxide: about 3.1%. The selected ratio is 0.6, but it still needs to be added to the fog seal for testing. Thus, we chose 0.6 for the following experiment.

**Figure 12:**
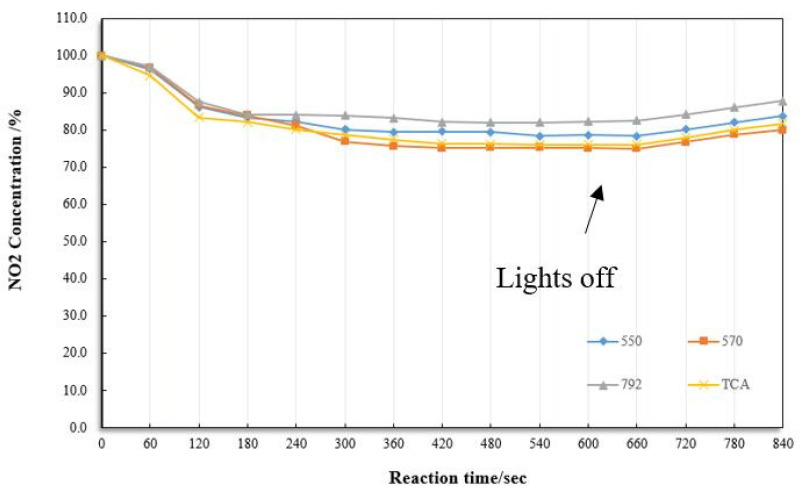
Photocatalytic efficiency of fog seal through different coupling agents.

**Figure 13:**
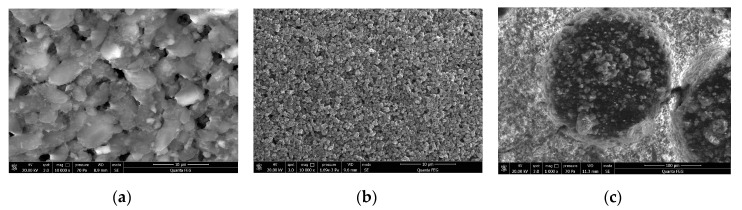
Through SEM: (**a**) pure fog seal; (**b**) pure titanium dioxide; (**c**) functionalized fog seal.

The photocatalytic efficiency of the composite through four coupling agents is shown in Figure 12. Some scholars found that TCA has a better modification effect on titanium dioxide [24]. From the figure, it can be seen that the degradation efficiency of KH570 and the titanium coupling agent is significantly better than the other two. The photocatalytic material’s degradation efficiency treated with the KH570 coupling agent is slightly higher than that of TCA. However, the composite material treated with the TCA can react quickly after facing nitrogen dioxide. It takes the shortest time to reach the maximal photocatalytic efficiency, which can neglect the slight difference in degradation efficiency compared to KH570. SEM images of pure fog seal, pure titanium dioxide, and functionalized fog seal modified by TCA are shown in Figure 13. Figure 13a illustrates the fog seal material, which is mainly composed of emulsified bitumen sand and water, forming several groups. The pure titanium dioxide particles have a particle size of 30 nanometers (as shown in Figure 13b). After mixing with TiO_2_ and active carbon, almost the entire active carbon group was covered by TiO_2_ adhering fog seal (as shown in Figure 13c). The composite material can be well dispersed in the fog seal. Thus, we chose the TCA for the next experiment.

**Figure 14:**
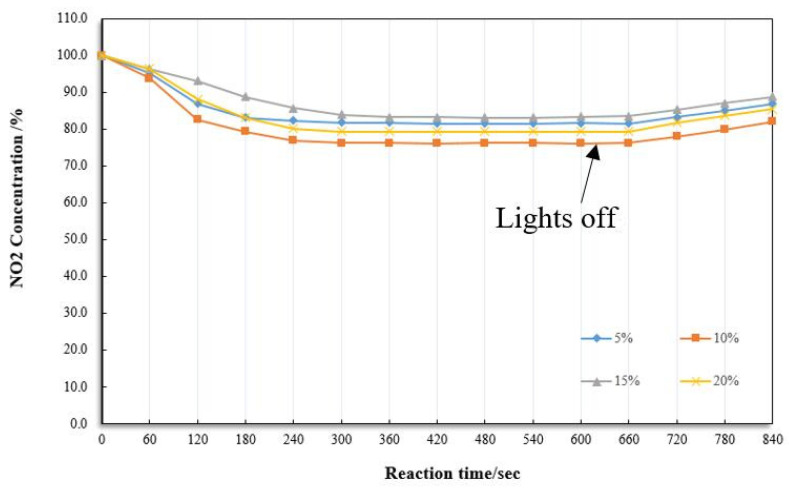
Degradation efficiency under different dosages.

We used “0.6” as the composite material ratio. Using modified TCA, we repeated the steps in Section 2.1 and added the 15 g fog seal. Figure 14 below shows the degradation efficiency of the fog seal in four proportions. In comparison of four doses, they are 5%, 10%, 15%, 20%, respectively. The maximum photocatalytic of efficiency was 18.6%, 23.9%, 16.9%, and 20.6%, respectively. When the photocatalyst content is 10%, the degradation efficiency and maximum degradation efficiency are higher than the other three. Therefore, the selected composite material dosage is 10% of the fog seal.

### 4.2. BPN Value

**Figure 15:**
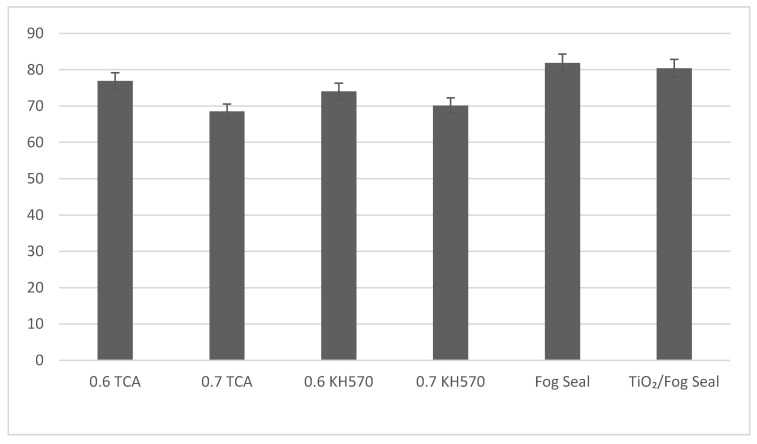
BPN value of different composite materials.

The BPN (British pendulum number) values were more than 65 for all the samples, which show good skid resistance. In particular, the BPN value of the Sand-fog seal is around 81. When the ratio of active carbon to titanium dioxide is 0.6 and TCA is used as a coupling agent to add to the fog seal, the BPN value is around 78 (as shown in Figure 15).

### 4.3. Outdoor Degradation Performance

After opening the traffic for five months, we conducted outdoor experiments. The experiment selected eight locations on the photocatalytic road and the original road for nitrogen oxide collection. After chemical analysis, the photocatalytic road surface’s nitrogen oxide content was less than about 31% compared to the ordinary road surface.

## 5. Discussion

This study suggests the optimal ratio of Sand-fog seal material with photocatalytic function. When the rate of active carbon to titanium dioxide is 0.6, it is modified by the titanate coupling agent and added to the fog seal at a dosage of 10%. The data show that pure titanium dioxide is superior to composite materials in terms of degradation growth rate and maximum degradation efficiency. That may be because a minimum of active carbon particles can guide pollutants to the titanium dioxide molecules’ surface for catalytic degradation. However, when the amount is large, the active carbon will adhere to the titanium dioxide’s body to prevent it from contacting the light source and inhibit the reaction from proceeding. In this study, no matter what proportion the composite materials account for, the inhibitory effect of the photocatalysis is more significant than their combined effect. When the composite material ratio is 0.6, its degradation efficiency is only slightly lower than that of pure titanium dioxide. Modifiers can improve titanium dioxide molecules’ surface properties to improve the compatibility between inorganic powder and organic phase liquid (asphalt). However, the composite material’s optimal degradation efficiency modified by the titanate coupling agent is slightly less than that of KH570. Its degradation rate is better than that of the other three coupling agents, considering the vehicle’s limited time to stay on the road after they are exhausted. Therefore, such a coupling agent was selected for further research. In this study, 10% is the optimal dosage. It is worth mentioning that when the dose is 10%, the mass of titanium dioxide and active carbon per 0.01 square meter is precisely 1, 0.6 g. This is the same as the number of the ratio selection of composite. It also indirectly shows that the fog seal will spoil almost half of the photocatalytic efficiency (51.9%, water as the carrier, while 23.9%, fog seal). In a specific area, the degradation efficiency is not related to the photocatalyst content linearly. Therefore, whether it is an indoor or outdoor experiment, only surface spreading needs to be considered. In this experiment, the calculated amount of composite material is about 1.6 g/0.01 m^2^.

The BPN values were more than 60 for all the samples. In particular, the BPN value of the Sand-fog seal is around 81. When the ratio of active carbon to titanium dioxide is 0.6 and TCA is used as a coupling agent to add to the fog seal, the BPN value is around 78 (as shown in Figure 14). This material has excellent skid resistance performance. From the comparison of active carbon content, the more active the carbon content, the more the skid resistance performance loss, which may be related to titanium dioxide or active carbon’s physical properties. In outdoor experiments, a significant photocatalytic reaction occurred. Considering the presence of this experiment on road surrounding farmland and fertilizer plants, nitrogen fertilizer may affect data collection. In automobile exhaust, there are still unburned HC or sulfur dioxide emissions. They may be adsorbed on the road through the physical properties of active carbon. However, these substances are difficult to be dissolved in water or easily washed away by rain.

## 6. Conclusions

This study combines titanium dioxide and active carbon in the Sand-fog seal. By coating material to the glass and placed it in a mold, it finds the optimal ratio. This device can instantly react on the effect of nitrogen oxide degradation. In engineering practice experiments, the research was carried out an outdoor experiment on the experimental road in Tianjin, China. The following points are the major findings of this study.

The synergistic effect of active carbon and titanium dioxide is less than the antagonistic effect. Active carbon can guide pollutants to the surface of titanium dioxide for photocatalytic reactions under its adsorption capacity. However, active carbon absorbs the light source and hinders the reaction. When the experimental temperature is 30 °C, the relative humidity is 30%, and the ratio of activated carbon to titanium dioxide is 0.6, the removal rate of nitrogen dioxide is the highest. In a specific area, the degradation efficiency is not related to the photocatalyst content linearly. TCA can well disperse titanium dioxide into the Sand-fog seal through SEM analysis and road nitrogen oxide detection.

Adding titanium dioxide and active carbon to the Sand-fog seal will not significantly impact the skid resistance performance. In outdoor experiments, the photocatalytic reaction can be detected. The materials can be used in engineering practice. In addition, it can be sprayed onto the surface repeatedly to maintain its photocatalytic function.

In future research, the impact of environmental factors on composite materials will be further considered by changing the temperature, humidity, and flow rate to explore the impact of its photocatalytic efficiency. It will promote special coatings of photocatalytic materials and specifically will be improved for road surfaces to minimize photocatalytic efficiency loss. There is a lack of suitable chemical or physical methods that have been found to detect active carbon adsorption quality. More experiments will be conducted to support the adsorption effect in the future.

## 7. Patents

This article involves two patents, which has now been obtained. Number: CN211043271U and CN201911098265.9.

## Figures and Tables

**Table 1:** Basic properties of anatase nano TiO_2_.

Project	Units	Indicators
Specific surface area	m^2^/g	≥280
Particle size	nm	30
Purity	%	≥99.9
Bulk density	g/cm^3^	0.2–0.3

**Table 2:** Basic properties of active carbon.

Project	Units	Indicators
Specific surface area	m^2^/g	900–1100
Purity	%	≥99.9
Filling density	g/cm^3^	0.45–0.55

**Table 3:** Physical properties of Sand-fog seal material.

Project	Units	Indicators
Proportion of 25 °C	-	≥1.18
Dry time	h	≤8
Volatile	-	Maximum loss of 10% weight

**Table 4:** The basic information of the four coupling agents.

Model	Chemical Name	Molecular Formula	Commercial Name
KH550	3-Aminopropyltriethoxysilane	C_9_H_23_NO_3_Si	Union Carbide Corporation: A-1100
KH570	3-Glycidoxypropyltrimethoxysilane	C_10_H_20_O_5_Si	Union Carbide Corporation: A-174
KH792	[3-(2-Aminoethyl)aminopropyl]trimethoxysilane	C_8_H_22_N_2_O_3_Si	Union Carbide Corporation: A-1120
TCA	Isopropyl tri(dioctylpyrophosphate) titanate	C_16_H_37_O_7_P_2_	Kenrich Corporation: KR-38S

**Table 5:** Materials component of ratios.

Ratio	0	0.1	0.3	0.5	0.6	0.7	1
Active Carbon (g)	0	0.1	0.3	0.5	0.6	0.7	1
Titanium Dioxide (g)	1	1	1	1	1	1	1
